# Le Miserable

**Published:** 1868-10

**Authors:** C. M. Wright


### LE MISERABLE.

BY C. M. WRIGHT.

There are some men who were never young. There are others who have forgotten that they were ever young, and strange to say, there are men practicing Dentistry; and other professions for whom the world smiles now, who have forgotten "the first five years of starvation" of professional life. These men can not sympathize with, nor give encouragement to young men, except by their present success and self-assurance, and their happy condition of forgetfulness. The young Dentist may take some comfort from these, and flatter himself that he too may some day forget to-day, and feel as selfishly independent, and as completely self-satisfied—he too, may some day pat the rising geniuses on the head, and with as kindly a condescension bid them "fear not little ones; have patience and all will be well." Can they have suffered as the young do now? Heavens! how terrible becomes that little word *wait.* "You must learn to *wait*, and in ten or twenty years you will begin to find that you are building up a practice, and that you are learning to walk alone." Ten years, after it has passed, is nothing. Ten years ago to a man who has lived three score years, is as but a day, or a month since, but the prospect of ten years of inactivity, of waiting, to a man who has lived but two or three times ten years, and whose blood is full of fire, whose muscles ache for a wrestle for dame fortunes' smile, whose brain is all activity, whose nerves buoy him up so that he seems to walk on air—ten years to him, I say, of crawling or waiting—well, the very idea is crushing. The young Dentist does not know the ways of the world. He will not admit the thought of this period of infancy, on his calculations to obtain success; so he opens an office in high spirits—fixes it up to the best of his means—prepares to do any amount of work—hangs out his name in gold letters, and after assuming all the airs of a busy man outside of his office, walks in and waits. He has, like the spider, spread his web, and he now *waits* for flies. If he is in a city, hundreds of people pass his office daily. Hundreds of eyes are cast on his sign, (the poor fellow sees this, and chuckles to himself behind his blinds) but he will yet learn that the large majority in a city "have eyes, but they see not." He waits. Days pass. No one rings his bell. He begins to think something must be wrong with the pull, and goes out stealthily to try it himself. The bell tinkles mockingly, so that is all right. He waits, weeks pass, and he begins to be surprised that people are so very careless of him. Bills are presented for rent, for gas, for water, for bread. His outgo is fine—there is no income. Advertising agents call on him and convince him that unless he lets people know that he is prepared to serve them, they will never find him out. He admits his card into the advertising columns, and reads it himself every day carefully. Why do not others see it? Months pass, and much of his courage and bright looks and joyous tones have disappeared, together with a good share of his little pile of money. He begins to mope about his office. He tires of his easy chairs. He gets sick of reading. His studies become irksome. The hours are like days at a country railroad station. Then his stomach gets disordered. His liver becomes deranged in its functions and the blue devils visit him daily. The shadow of melancholy rests on his heart and he is miserable. In vain he tries to fling off this shadow. In vain he tries to bid his blue companion good bye. He calls upon his reason to help him. He whistles and sings, but his own voice startles him. Then a sense of desperation seizes him, and he resolves to make another effort to induce some of the multitude to come in, and prove for themselves that he is a rising man. He advertises again, and this time allows more than a simple, unattractive card. He spends hours and hours in writing an advertisement that will suit, and finds this to be the most difficult style of composition. The question arises, "how can I attract people to come to me that I may live, and yet not violate the code of ethics?" Ah! he sweats over that question. At length his card appears in the daily papers. He trembles a little as he reads it; but when he is dead cut on the street by an old practitioner, and once friend, he feels indignant, and knows he is not a quack, and is willing to defend himself of the charge. Foolish fellow! His quiet, inactive, gloomy life has made him over sensitive. He broods over his own affairs too much. The cut was entirely accidental, and not one man that he cares for in the profession ever gave his card a thought.

But his advertisement has been seen, for his bell rings and a patient enters. "Doctor I've seen your card in the papers and I called to inquire your prices." He replies modestly, and is answered indignantly, "Pooh! why Dr. Seventy-five-years-old don't charge any more than you do." He loses that patient and many more. Then he feels that though he has a stock of Dental knowledge and skill on hand there is no demand for it, and he having studied Whately while in college determines to act on economical principles. He offers to sell his brains, and sweat, and hands cheap. His bell rings oftener now, but the class that calls brow-beat, bally-rag, jew, insult and torture him, and he wonders why the good Lord ever permitted him to enter a profession. Why have I devoted years to prepare myself for such misery? Why did I not go to blacksmithing, or plowing, or to day labor? Oh! my readers his heart is heavy, and he wonders whether the sleek looking, contented old men really did go through all this. He wants sympathy or encouragement, and he goes to successful men in his own and other professions with his tale of woe. They laugh at his troubles, give him an empty compliment, yawn and say "wait." At this point the temperament decides the course of the poor fellow. One will moan and take a drink, and with a sad voice and a tearful eye inquire the price of charcoal. Another swears and quits the profession. Our poor fellow grits his teeth (perhaps he swears deep, but not loud), clenches his fists, assumes a theatrical position, after the legitimate drama manner and exclaims: "Come on ye fates and furies. I'll not give way to you. On this ground I'll stand and fight ye. Pile up ye debts and duns. I'll laugh at you. Come rags and starvation, I'll try you too, but here I'll stand with my armor on, and midst the crush of matter and the wreck of worlds, I'll die, or live, and *wait* for twenty years, but I *will* win the palm of victory." My friends will this one win? And if he does, what will he win? What but the satisfaction of knowing that he is devoting life to a philanthropic profession—that he is doing good daily—that he earns his bread in the sweat of his brow—that his position in society is the "professional class"—that he is a gentleman in heart and manners—that he is a good citizen and a quiet student—that he will never spoil his children by affording them the means of living without industry. What else will be gain? He may have friends. He will get old in harness, and younger men will push him aside, and he'll die. Is not this all? Well, where can he do better, or where can he fare worse?

